# Effect of Corticosteroids on C-Reactive Protein in Patients with Severe Community-Acquired Pneumonia and High Inflammatory Response: The Effect of Lymphopenia

**DOI:** 10.3390/jcm8091461

**Published:** 2019-09-13

**Authors:** Antoni Torres, Adrian Ceccato, Miquel Ferrer, Albert Gabarrus, Oriol Sibila, Catia Cilloniz, Raúl Mendez, Rosario Menendez, Jesus Bermejo-Martin, Michael S. Niederman

**Affiliations:** 1 Department of Pneumology, Institut Clinic de Respiratori, Hospital Clinic of Barcelona-Institut d’Investigacions Biomèdiques August Pi i Sunyer (IDIBAPS), University of Barcelona (UB), Villarroel 170, 08036 Barcelona, Spain; aceccato@clinic.cat (A.C.); gabarrus@clinic.cat (A.G.); catiacilloniz@clinic.cat (C.C.); 2 Centro de Investigación Biomedica En Red-Enfermedades Respiratorias (CibeRes, CB06/06/0028), 28029 Madrid, Spain; rosmenend@gmail.com (R.M.); jfbermejo@saludcastillayleon.es (J.B.-M.); 3 Servei de Pneumologia, Hospital de la Santa Creu i Sant Pau, Sant Antoni Maria Claret 167, 08025 Barcelona, Spain; OSibila@santpau.cat; 4 Servicio de Neumología, IIS/Hospital Universitario y Politécnico La Fe, Avinguda de Fernando Abril Martorell, n° 106 Valencia, CIBERES, 46026 València, Spain; rmendezalcoy@gmail.com; 5 PhD program in Medicine and Traslational Research, University of Barcelona, Gran Via de les Corts Catalanes, 585, 08007 Barcelona, Spain; 6 Group For Biomedical Research in Sepsis (BioSepsis), Instituto de Investigación BIomédica de Salamanca (IBSAL), 37007 Salamanca, Spain; 7 Division of Pulmonary and Critical Care Medicine, Weill Cornell Medical College, New York, NY 10065, USA; msniederman@gmail.com

**Keywords:** pneumonia, corticosteroids, inflammatory response

## Abstract

Background: Lymphopenic patients with community-acquired pneumonia (CAP) have shown high mortality rates. Corticosteroids have immunomodulatory properties and regulate cytokine storm in CAP. However, it is not known whether their modulatory effect on cytokine secretion differs in lymphopenic and non-lymphopenic patients with CAP. Therefore, we aimed to test whether the presence of lymphopenia may modify the response to corticosteroids (mainly in C reactive protein (CRP)) in patients with severe CAP and high inflammatory status). Methods: A post hoc analysis of a randomized controlled trial (NCT00908713) which evaluated the effect of corticosteroids in patients with severe CAP and high inflammatory response (CRP > 15 mg/dL). Patients were clustered according to the presence of lymphopenia (lymphocyte count below 1000 cell/mm^3^). Results: At day 1, 35 patients (59%) in the placebo group presented with lymphopenia, compared to 44 patients (73%) in the corticosteroid group. The adjusted mean changes from day 1 showed an increase of 1.19 natural logarithm (ln) cell/mm^3^ in the corticosteroid group and an increase of 0.67 ln cell/mm^3^ in the placebo group (LS mean difference of the changes in ln (methylprednisolone minus placebo) 0.51, 95% CI (0.02 to 1.01), *p* = 0.043). A significant effect was also found for the interaction (*p* = 0.043) between corticosteroids and lymphopenia in CRP values at day 3, with lower values in patients without lymphopenia receiving corticosteroids after adjustments for potential confounders. Conclusion: In this exploratory post hoc analysis from ramdomized controlled trial (RCT) data, the response to corticosteroids, measured by CRP, may differ according to lymphocyte count. Further larger studies are needed to confirm this data.

## 1. Introduction

Lymphopenic patients with community-acquired pneumonia (CAP) have shown high mortality rates [1]. The presence of prior conditions leading to immunosuppression, the recruitment/escape of these cells to sites of inflammation/infection, and/or apoptotic phenomena may explain the presence of low lymphocyte counts in patients with CAP [2]. Corticosteroids have immunomodulatory properties and regulate cytokine storm in CAP. However, it is not known whether their modulatory effect on cytokine secretion differs in lymphopenic and non-lymphopenic patients with CAP. Therefore, we aimed to test whether the presence of lymphopenia may modify the response to corticosteroids (mainly in C reactive protein (CRP)) in patients with severe CAP and high inflammatory status.

## 2. Methods and Patients

We performed a post hoc analysis of a randomized controlled trial [3] (NCT00908713) which evaluated the effect of corticosteroids in patients with severe CAP and high inflammatory response (CRP > 15 mg/dL). We clustered patients according to the presence of lymphopenia (lymphocyte count below 1000 cell/mm^3^). We reported the number and percentage of patients for categorical variables and the median (interquartile range) for continuous variables. Categorical variables were compared using the x^2^. Continuous variables were compared using the nonparametric Kruskal-Wallis test. Pairwise comparisons were carried out via the Bonferroni method in order to control the experiment-wise error rate. We fitted analysis of covariance (ANCOVA) model [4,5] to analyze the change from baseline in lymphocyte counts at day 3, adjusting for the treatment, PSI risk class, year of recruitment, and center. Each treatment effect was estimated by the Least Square (LS) mean, its standard error (SE), and 95% confidence interval (CI). Lymphocyte counts were log-transformed to fit the ANCOVA model. We also analyzed the CRP values at day 3 by means of ANCOVA models [4,5], adjusting for the baseline values, treatment, lymphocyte counts, lymphocyte counts x treatment interaction, PSI risk class, year of recruitment, and centre. Each treatment effect was estimated by the LS mean, its SE, and 95% CI. CRP values were log-transformed to fit the ANCOVA model. Further information on our study is provided elsewhere [3].

## 3. Results

Of the 120 patients, 57 received placebo and 54 received corticosteroids in the per protocol (PP) population. On day 1, 35 patients (59%) in the placebo group presented with lymphopenia compared to 44 patients (73%) in the corticosteroid group.

Baseline characteristics, CRP values, and clinical outcomes are summarized in Table 1. At day 3 adjusted mean increased from day 1 in natural logarithms (ln) of lymphocyte count for those with lymphopenia. This was higher for corticosteroid than for placebo patients. The adjusted mean changes from day 1 showed an increase of 1.19 ln cell/mm^3^ in the corticosteroid group and an increase of 0.67 ln cell/mm^3^ in the placebo group (LS mean difference of the changes in ln (methylprednisolone minus placebo) 0.51, 95% CI (0.02 to 1.01); *p* = 0.043).

**Figure 1:**
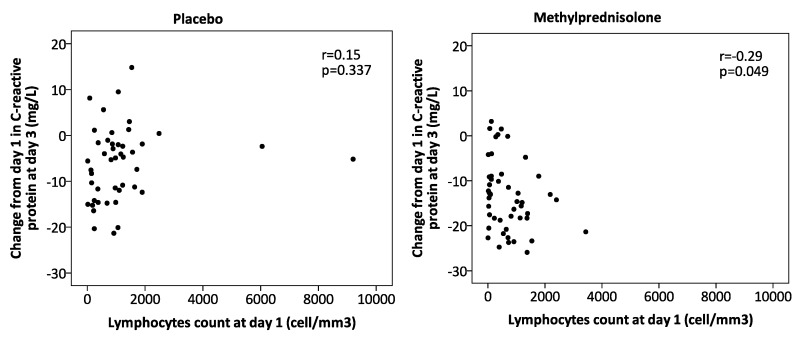
Correlation between the change from day 1 in C-reactive protein at day 3 and lymphocyte count at day 1 for the per protocol population.

A significant effect was also found for the interaction (*p* = 0.043) between corticosteroids and lymphopenia in CRP values at day 3 (Table 1), with lower values in patients without lymphopenia receiving corticosteroids after adjustments for potential confounders. Moreover, in patients who received corticosteroids, a negative correlation between lymphocyte count and the value of change in CRP was found (*r* = −0.29 *p* = 0.049). This correlation was not significant in patients who received placebo (Figure 1). Treatment failure rates did not differ between the four groups.

## 4. Discussion

Our study found that patients who received corticosteroids presented differences in the systemic anti-inflammatory effect as measured by CRP at day 3, with variations related to the presence or absence of lymphopenia. However, we did not find a clinical impact on treatment failure because the analysis was underpowered for this outcome. Previous studies have shown that levels of CRP at day 3 are associated with treatment failure in CAP [6]. Corticosteroids have immunomodulatory activity through the inhibition of NF-kappaB activity and activator protein 1, both of which are transcription factors that activate immunoregulatory genes [7] and might decrease the rates of treatment failure. Recently, a post hoc analysis in an RCT of corticosteroids in septic shock also showed a differential response according to previously described transcriptomic sepsis response signatures [8,9].

In this post hoc analysis, patients who received corticosteroids showed higher increases in lymphocytes at day 3, contrary to the expectations that corticosteroids might produce lymphopenia [10] due to apoptosis or by a redistribution of recirculating lymphocytes at an early stage. A greater response from bone marrow and redistribution from lymph nodes induced by corticosteroids may explain the increase at day 3. In spite of these results, the clinical impact of lymphocyte response to corticosteroids remains unknown.

There are two main limitations in our study. First, it is a post hoc analysis of a previous RCT without a specific sample size calculation for the current investigation purpose. Second, there were disbalances in the basal CRP with lower values in patients from the placebo group and lymphopenia compared with the other groups. However, and concerning this second point, what really matters when evaluating a biomarker is the delta differences, in this case between baseline and day 3 [11]. The major strength is the validity of data that proceeds from a RCT.

## 5. Conclusions

In conclusion, the response to corticosteroids, measured by CRP, may differ according to lymphocyte count. The low number of patients in each group does not allow an analysis of clinical outcomes. However, in view of our results, new studies are warranted to evaluate the effect of corticosteroids in non-lymphopenic CAP patients where higher benefits could be observed.

## Figures and Tables

**Table 1:** Baseline characteristics and outcomes of the intention-to-treat population (*n* = 119) and outcomes of the per protocol population (*n* = 111).

Intention-to-Treat Population	Placebo Patients with Lymphopenia (*n* = 35)	Placebo Patients without Lymphopenia (*n* = 24)	Methylprednisolone Patients with Lymphopenia (*n* = 44)	Methylprednisolone Patients without Lymphopenia (*n* = 16)	*p* value
Age, median (IQR), years	66 (44; 82)	75.5 (62.5; 81)	75 (50.5; 81.5)	63.5 (55; 75)	0.331
Male sex, *n* (%)	22 (63)	17 (71)	25 (57)	9 (56)	0.682
Current smoker, *n* (%)	10 (29)	7 (29)	9 (20)	6 (38)	0.581
Pre-existing comorbid conditions, *n* (%)					
Diabetes mellitus	7 (20)	6 (25)	8 (18)	2 (13)	0.796
Chronic pulmonary disease	5 (15)	7 (29)	6 (14)	1 (7)	0.213
Hypertension	12 (35)	12 (50)	17 (39)	5 (31)	0.582
History of malignancy	2 (6)	6 (25)	3 (7)	0 (0)	0.023
Ischemic heart disease	3 (9)	6 (25)	9 (20)	3 (19)	0.372
Pneumonia Severity Index score, median (IQR)	117.5 (100; 136)	103 (85; 130)	111 (85; 138)	107 (80.5; 130)	0.718
APACHE II, median (IQR)	16 (11; 20)	16 (14; 23)	14 (10; 16)	16 (14; 20)	0.151
SOFA score, median (IQR)	4 (3; 5)	5 (5; 7)	4 (2; 6)	3.5 (1.5; 5)	0.307
Outcomes					
Treatment failure, *n* (%)	10 (29)	8 (33)	5 (11)	3 (19)	0.127
In-hospital mortality, *n* (%)	5 (14)	4 (17)	5 (11)	1 (6)	0.778
CRP at day 1, mg/L	268.5 (233.4; 295.4)	181.6 (163.2; 244.4)	259.6 (197.6; 289.8)	282.8 (242.5; 292.7)	0.018 ^ae^
CRP at day 1, >500 mg/L	0	0	3 (7)	0	0.153
CRP at day 1, >1000 mg/L	1 (3)	1 (5)	4 (10)	0	0.424
CRP at day 3, mg/L	197.7 (1134; 246)	144.6 (106.7; 228.5)	100.8 (62.8; 151.9)	107.9 (69.2; 125.6)	0.007 ^b^
Change from baseline in CRP at day 3, mg/L	−79.3 (−146.3; −18.7)	−36.6 (−108.5; 4.2)	−128.3 (−185.5; −80.7)	−152.3 (−183.1; −130.5)	<0.001 ^cde^
Per protocol population	Placebo patients with lymphopenia (*n* = 33)	Placebo patients without lymphopenia (*n* = 24)	Methylprednisolone patients with lymphopenia (*n* = 40)	Methylprednisolone patients without lymphopenia (*n* = 14)	
Outcomes					
Treatment failure, *n* (%)	8 (24)	8 (33)	3 (8)	2 (14)	0.059
In-hospital mortality, *n* (%)	3 (9)	4 (17)	3 (8)	0 (0)	0.360
CRP at day 1, mg/L	269.5 (233.4; 295.4)	181.6 (163.2; 244.4)	254.7 (197.6; 289.8)	283.2 (251.4; 292.7)	0.009 ^ae^
CRP at day 3, mg/L	169.7 (113.4; 246)	144.6 (106.7; 228.5)	100.8 (50.3; 151.9)	107.9 (69.2; 125.6)	0.007 ^b^
Change from baseline in CRP at day 3, mg/L^f^	−79.3 (−146.3; −18.7)	−36.6 (−108.5; 4.2)	−128.3 (−187.7; −85.2)	−152.3 (−183.1; −130.5)	<0.001 ^cde^

Abbreviations: APACHE, Acute Physiology and Chronic Health Evaluation, CRP, C reactive protein, IQR, interquartile range; SOFA, Sequential Organ Failure Assessment. Bold indicates significant p values. Percentages calculated on non-missing data. ^a^ *p* < 0.05 for comparison between the placebo patients with lymphopenia group and placebo patients without lymphopenia group. ^b^ *p* < 0.05 for comparison between the placebo patients with lymphopenia group and methylprednisolone patients with lymphopenia group. ^c^ *p* < 0.05 for comparison between the placebo patients with lymphopenia group and methylprednisolone patients without lymphopenia group. ^d^ *p* < 0.05 for comparison between the placebo patients without lymphopenia group and methylprednisolone patients with lymphopenia group. ^e^ *p* < 0.05 for comparison between the placebo patients without lymphopenia group and methylprednisolone patients without lymphopenia group. ^f^ The analysis based on the ANCOVA model for CRP at day three as response and with treatment group, lymphopenia, treatment group and lymphopenia interaction, severity (PSI score), year and centre of enrolment as factors along with baseline CRP as covariate estimated a LS mean (95% CI) of 87.4 mg/dL (55.1 to 138.5) in the placebo patients with lymphopenia group, a LS mean (95% CI) of 84.4 mg/dL (55.7 to 128.1) in the placebo patients without lymphopenia group, a LS mean (95% CI) of 147.4 mg/dL (86.6 to 266.4) in the methylprednisolone patients with lymphopenia group, and a LS mean (95% CI) of 60.8 mg/dL (31.7 to 116.4) in the methylprednisolone patients without lymphopenia group. The *p* values obtained from the ANCOVA model were 0.021 for the corticosteroid effect, 0.660 for the lymphopenia effect, and 0.043 for the treatment group and lymphopenia interaction effect.
